# Very small embryonic-like stem cells are involved in regeneration of mouse pancreas post-pancreatectomy

**DOI:** 10.1186/scrt494

**Published:** 2014-09-02

**Authors:** Deepa Bhartiya, Akash Mundekar, Vaibhav Mahale, Hiren Patel

**Affiliations:** Stem Cell Biology Department, National Institute for Research in Reproductive Health (ICMR), Mumbai, 400012 India

## Abstract

**Introduction:**

Despite numerous research efforts, mechanisms underlying regeneration of pancreas remains controversial. Views are divided whether stem cells are involved during pancreatic regeneration or it involves duplication of pre-existing islets or ductal cells or whether pancreatic islet numbers are fixed by birth or they renew throughout life. Pluripotent embryonic stem (ES) and induced pluripotent stem (iPS) cells have been used by several groups to regenerate diabetic mouse pancreas but the beneficial effects are short-lived. It has been suggested that cells obtained after directed differentiation of ES/iPS cells resemble fetal and not their adult counterparts; thus are functionally different and may be of little use to regenerate adult pancreas. A novel population of pluripotent very small embryonic-like stem cells (VSELs) exists in several adult body tissues in both mice and humans. VSELs have been reported in the mouse pancreas, and nuclear octamer-binding transcription factor 4 (OCT-4) positive, small-sized cells have also been detected in human pancreas. VSELs are mobilized into peripheral blood in streptozotocin treated diabetic mice and also in patients with pancreatic cancer. This study aimed to evaluate whether VSELs are involved during regeneration of adult mouse pancreas after partial pancreatectomy.

**Methods:**

Mice were subjected to partial pancreatectomy wherein almost 70% of pancreas was surgically removed and residual pancreas was studied on Days 1, 3 and 5 post-surgery.

**Results:**

VSELs were detected in Hematoxylin and Eosin stained smears of pancreatic tissue as spherical, small sized cells with a large nucleus surrounded by a thin rim of cytoplasm and could be sorted as LIN-/CD45-/SCA-1+ cells by flow cytometry. Results reveal that although neutrophils with multi-lobed nuclei are mobilized into the pancreas on day 1 after pancreatectomy, by day 5 VSELs with spherical nuclei, high nucleo-cytoplasmic ratio and nuclear OCT-4 are mobilized into the residual pancreas. VSELs undergo differentiation and give rise to PDX-1 and OCT-4 positive progenitors which possibly regenerate both acinar cells and islets.

**Conclusions:**

Results provide direct evidence supporting the presence of VSELs in adult mouse pancreas and their role during regeneration. VSELs are an interesting alternative to ES/iPS cells to regenerate a diabetic pancreas in future.

## Introduction

Despite decades of research, the mechanism underlying regeneration of adult pancreas remains controversial [1, 2]. Bouwens and colleagues concluded in their recent review that although the pancreas has long been known to have huge potential to regenerate, it is still not evident whether the pancreas houses stem cells for regeneration or not [3]. No consensus exists as to whether regeneration occurs by duplication of pre-existing cells or pancreatic ductal stem cells are involved. Wang and colleagues provided evidence via differential BrdU uptake by the beta cells and ductal epithelium in the pancreas after pancreatectomy that beta cells do not form from pre-existing islets [4]. Xu and colleagues provided further evidence against the concept of re-duplication of pre-existing islets by showing the existence of novel multipotent progenitors in mouse pancreas that may be responsible for regeneration of beta islet cells [5], and their findings have huge translational value to treat diabetes. Understanding the underlying mechanisms of pancreatic regeneration post pancreatectomy becomes crucial and an urgent quest exists to find adult pancreas stem cells. This kind of understanding will help to tackle the increasing magnitude of diabetes that has become a global epidemic. At present, there are approximately 346 million adult diabetic patients worldwide. By 2030 the number of diabetic patients is expected to reach 4 billion, and China and India are leading diabetes prevalence in the world [6].

Stem cells have captured the fascination of one and all because of their possible applications in regenerative medicine. The stem cells are broadly classified as pluripotent (embryonic stem (ES), induced pluripotent stem (iPS)) cells and tissue-specific adult multipotent or unipotent stem cells. Ratajczak’s group proposed the existence of an entirely novel group of pluripotent stem cells in adult body organs [7] termed very small embryonic-like stem cells (VSELs), and their very existence in adult body organs makes redundant the need to grow other pluripotent stem cells (ES or iPS cells) in a Petri dish for regenerative medicine. VSELs (LIN^–^/CD45^–^/SCA-1^+^) are hypothesized to be derived from the late migrating primordial germ cells and deposited in various body organs during early embryonic development [8–10], are mobilized under disease conditions [11–16] and are hypothesized to be possible embryonic remnants responsible for various cancers in adult life [17]. As expected from other pluripotent stem cells (ES and iPS cells), VSELs have the ability to self-renew and differentiate into three lineages in humans [18] as well as in mice [19]. Unlike ES and iPS cells, however, VSELs do not divide rapidly in culture, do not complement blastocyst development and do not form teratoma on being injected in immunocompromised mice. This inability of VSELs is due to a novel epigenetic mechanism of imprint erasure on paternally imprinted differentially methylated regions (DMRs) (H19-Igf2, RasGRF1) [9] and as a result they remain relatively quiescent and most probably undergo asymmetric cell divisions compared with ES cells, which undergo rapid symmetric divisions *in vitro*. Besides maintaining normal homeostasis, VSELs have been implicated to play an important role in regeneration [20, 21]. We have reported them in cord blood, bone marrow and adult testis and ovary [22, 23]. We have further observed that VSELs survive chemotherapy in both mouse ovary and testis, in agreement with an earlier report that VSELs survive in mouse bone marrow after radiotherapy whereas the hematopoietic stem cells are destroyed [24], and can restore gonadal function when appropriate conditions are provided (DB, 2014). The main reason why these stem cells have remained elusive until now is their very small size, as a result of which they are easily and unknowingly discarded during processing. Here we provide morphological and additional evidence about their existence and homing ability into the mouse pancreas after pancreatectomy. Hopefully, observing nuclear OCT-4-positive VSELs in a morphological context in the blood vessels and their presence in the newly formed lobes of pancreas that regenerate by day 5 post pancreatectomy in mice along with progenitors which colocalize OCT-4 and pancreas duodenum homeobox-1 (PDX-1) by various technical approaches (immunolocalization, confocal microscopy and reverse transcription-polymerase chain reaction) will convince the scientific community about the existence and potential of pancreatic VSELs.

## Methods

The study was approved by the Institute Animal Ethics Committee (IAEC/NIRRH 19/13) and was carried out using in-house bred Swiss mice in the experimental animal facility. Mice were housed in a temperature-controlled and humidity-controlled room on a 12-hour light/12-hour darkness cycle with free access to food and water. Swiss mice 8 to 10 weeks old were used for partial pancreatectomy as described earlier [25]. Residual pancreas was harvested on days 1, 3 and 5 after surgery for histology, immunolocalization and RNA extraction. Pancreatic tissue was also appropriately processed to obtain cell suspensions for immunofluorescence and flow cytometry studies.

### Histology

The pancreas was removed after partial pancreatectomy, formalin fixed, processed and embedded in paraffin using standard protocols. Then 5 μm thick sections of the embedded pancreatic tissue were prepared and stained with hematoxylin and eosin (H & E) to study the histo-architecture. The representative areas were photographed using a 90i microscope (Nikon, Tokyo, Japan).

### Immunolocalization studies

Immunolocalization studies were carried out for OCT-4 (nuclear transcription factor reflecting a pluripotent state) and PDX-1 (nuclear transcription factor specific for pancreas development). Briefly, the paraffin sections were deparaffinized and hydrated and antigen retrieval was performed by boiling in saline sodium citrate buffer (pH 6) for 15 minutes. After cooling, the slides were washed with Tris buffer saline for 5 minutes followed by blocking with 10% serum and 1% bovine serum albumin in Tris buffer saline. Later the slides were incubated with primary antibody diluted with 1% bovine serum albumin in Tris buffer saline for 2 hours at room temperature (polyclonal OCT-4 antibody, 1:200 and polyclonal PDX-1, 1:500 antibody; both Abcam, Cambridge, UK). The detection was carried out using the anti-mouse Vecta ABC kit (Vector Laboratories, Burlingame, CA, USA) and diaminobenzidine (BioGenex, San Ramon, CA, USA). After counterstaining with hematoxylin, the slides were viewed under a microscope.

### Reverse transcription-polymerase chain reaction studies

Total RNA from the pancreas was extracted by standard protocol using Trizol (Invitrogen, Carlsbad, CA, USA). Briefly, 2 μg RNA without Dnase 1 treatment were used for cDNA synthesis. The reaction was carried out in a G-STORM thermocycler (Gene Technologies, Braintree, UK) and cDNA was synthesized using the Omniscript RT Kit according to the manufacturer’s instructions (Qiagen GmbH, Hilden, Germany). Then 1 mM random hexamer primers, 0.5 mM deoxynucleotide triphosphates, 5 units of Moloney murine leukemia virus reverse transcriptase, and 10 units of RNase inhibitor in a single-strength reaction buffer were used to synthesize the first strand of cDNA in a total volume of 20 μl. The primers and the RNA were incubated at 70°C for 2 minutes and chilled at 4°C for 5 minutes and then at 37°C for 1 hour. cDNA mix (2 μl) was amplified using 10 pmol each primer (Table 1), 1 unit of Taq DNA polymerase (Fermentas Life Sciences, Vilnius, Lithuania), 1.5 mm MgCl_2_ and 10 mM deoxynucleotide triphosphates in a 25 μl reaction volume in a G STORM thermocycler. Amplification was carried out for 35 cycles, with each cycle consisting of denaturation at 94°C for 1 minute, annealing at the specified temperature for each set of primers (Table 1) for 1 minute 30 seconds, and extension at 72°C for 2 minutes. The products were analyzed on 1.5% agarose gel stained with 0.5 mg/ml ethidium bromide (Bangalore Genei, Bangalore, India) and visualized under ultraviolet transillumination. The product size was approximated using a 100-base-pair DNA ladder (Bangalore Genei). The negative control did not include cDNA in the reaction mixture. RNA extraction and amplification from the pancreas was challenging because RNA degrades easily, the pancreas being rich in RNAses, and thus we were unable to perform quantitative reverse transcriptase polymerase chain reaction studies.

**Table 1:** **Primers used for reverse transcription**-**polymerase chain reaction studies**

Sr No	Gene	Primer sequence	Product size (base pairs)
1	*Oct-4A*	Forward: CCATGTCCGCCCGCATACGA	235
		Reverse: GGGCTTTCATGTCCTGGGACTTCT	
2	*Sox-2*	Forward: AGGAGTTGTCAAGGCAGAGAAGAGA	167
		Reverse: GCCGCCGCGATTGTTGTGATT	
3	*Oct-4*	Forward: CCTGGGCGTTCTCTTTGGAAAGGTG	177
		Reverse: GCCTGCACCAGGGTCTCCGA	
4	*Gapdh*	Forward: GTCCCGTAGACAAAATGGTGA	458
		Reverse: TGCAATGCTGACAATCTTGAG	

### Preparation of pancreatic cell suspension for various studies

Pancreatic cell suspension was prepared by digestion of tissue with collagenase V (1 mg/ml) for 15 minutes followed by filtration through a 40 μm filter. The cell suspension was centrifuged at 1000 × *g* for 10 minutes, fixed in 4% paraformaldehyde, washed in phosphate-buffered saline and then smears prepared on poly-l-lysine-coated slides. Normal pancreas smears were stained with H & E whereas day 5 smears were used for colocalization of OCT-4 and PDX-1. Smears were washed with phosphate-buffered saline, permeabilized with 0.3% Triton X followed by blocking for 2 hours, and then incubated overnight at 4°C with OCT-4 and PDX-1 antibodies. After washing with phosphate-buffered saline containing 0.5% bovine serum albumin and 0.5 mM ethylenediamine tetraacetic acid, smears were incubated with secondary antibodies (anti goat Alexafluor 488 for PDX-1 and anti-rabbit Alexafluor 568 for OCT-4) for 2 hours followed by washing, counterstained with 4′,6-diamidino-2-phenylindole, washed, mounted using Vectashield and sealed with nail polish. The slides were viewed under a laser scanning confocal fluorescent microscope (LSM 510-META; Zeiss, Jena, Germany) and representative fields were photographed.Cell suspension prepared by enzymatic digestion of normal pancreas was also subjected to flow cytometry to enumerate VSELs. The cells were stained with fluorescein isothiocyanate rat anti-mouse SCA-1 (1 μg/million cells, 553335; BD Pharmingen, San Diego, CA, USA), phycoerythrin rat anti-mouse CD45 (2 μg/million cells, 553081; BD Pharmingen) and allophycocyanin mouse lineage antibody cocktail (25 μl/ml cells, 51-9003632; BD Pharmingen). The stained cells were run on FACS Aria and the results were analyzed using FACS Diva software (BD Biosciences). Briefly, calibration beads of size 2 to 15 μm were used according to the manufacturer’s instructions (Life Technologies, Carlsbad, CA, USA) as reference for selecting cells ranging from 2 to 5 μm and to set the threshold accordingly.

**Figure 1:**
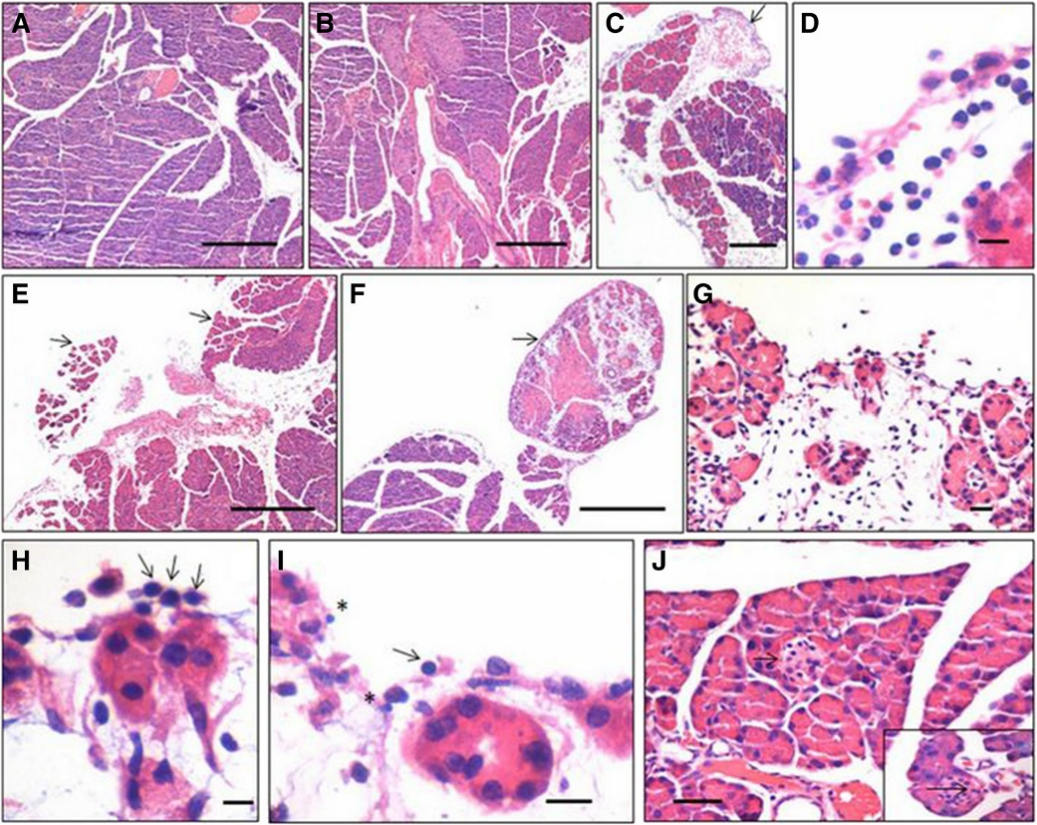
**Hematoxylin and eosin-stained sections of normal mouse pancreas and after partial pancreatectomy. (A)** Normal pancreas section showing the presence of acinar cells, ducts, blood vessels as well as islets. **(B)** Pancreas on day 3 post pancreatectomy showing prominent ‘hypertrophied’ islets, probably because they have to compensate for the islets lost due to pancreatectomy. **(C)** A prominent layer of coelomic epithelium (arrow) covers the pancreas post pancreatectomy. **(D)** On day 1 post surgery, most of the infiltrating leukocytes appear to be neutrophils with multilobed nuclei. **(E)**, **(F)** A large number of cells migrate under the coelomic epithelium layer; newly formed lobes can be visualized clearly (arrow). **(G)** Higher magnification of newly formed pancreatic lobe covered by coelomic epithelium. Note newly formed acinar cells aligned below the coelomic epithelium. **(H)**, **(I)** At higher magnification, acinar cells are clearly differentiating adjacent to coelomic epithelium and a few distinct spherical stem cells with minimal cytoplasm are also visualized. Two distinct sizes of stem cell are observed, including very small (asterisk) and slightly bigger (arrow) in size. **(J)** Small islets are clearly visualized in the newly forming pancreatic lobes. Presence of new islets in the regenerating region clearly suggests their neogenesis rather than re-duplication from surviving islets as per existing paradigm. Scale bar represents 20 μm.

## Results

### Histological changes post pancreatectomy

**Figure 2:**
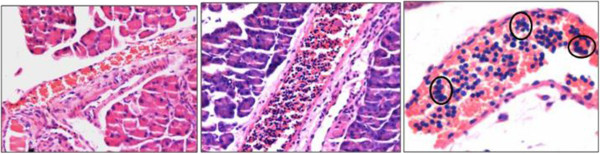
**Blood vessels in the pancreas. (A)** In normal pancreas, the blood vessels are full of red blood cells as expected. **(B)**, **(C)** Partial pancreatectomy results in a dramatic homing of cells clearly visualized in the blood vessels in large numbers on day 5. These cells are distinctly spherical, with high nucleocytoplasmic ratio and minimal cytoplasm, and also appear as clumps in places. This cell population is distinct from the neutrophils observed on day 1 (Figure 1D). The presence of these stem cells in clumps in places possibly reflects clonal and rapid expansion of stem cells with incomplete cytokinesis. This homing of stem cells probably represents the process of regeneration and is not an inflammatory response. Scale bar represents 20 μm.

Several groups have reported regeneration of pancreas after pancreatectomy and have implicated the involvement of pre-existing islet cells or neogenesis of endocrine cells from epithelial cells or from the ductal epithelium. Even prior streptozotocin (STZ) treatment does not inhibit pancreatic regeneration after pancreatectomy [25, 26]. The islets in surviving pancreatic tissue undergo extensive hypertrophy (Figure 1B) to compensate for the removed pancreas function compared with normal pancreas (Figure 1A). Post pancreatectomy, the pancreas surface becomes surrounded by coelomic epithelium (Figure 1C) and the region is infiltrated with blood cells resembling neutrophils with multilobed nuclei (Figure 1D) exemplifying inflammatory response on day 1 post pancreatectomy. New lobes of pancreas are formed and are easily distinguished from the old pancreas (Figure 1E,F) by days 3 to 5. New individual acinar cells are observed aligned along the coelomic epithelium as if they are differentiating along the epithelium and then push down to form the pancreatic lobes (Figure 1G,H,I). It was intriguing to note the presence of distinct spherical stem cells with high nucleocytoplasmic ratio of two distinct sizes in this region (Figure 1H,I). Newly formed islets were also observed in this region (Figure 1J). Compared with blood vessels in normal pancreas full of red blood cells (Figure 2A), a large number of cells with distinct morphology home into the pancreas on day 5 (Figure 2B,C). These cells were typically spherical in shape, had high nucleocytoplasmic ratio and darkly stained nuclei, and also present as small clusters in places (Figure 2C). These cells were distinct from the neutrophils with multilobed nuclei, suggestive of an inflammation observed on day 1 (Figure 1D), and indeed represent stem cell mobilization and an attempt by the body to regenerate the damaged pancreas.

**Figure 3:**
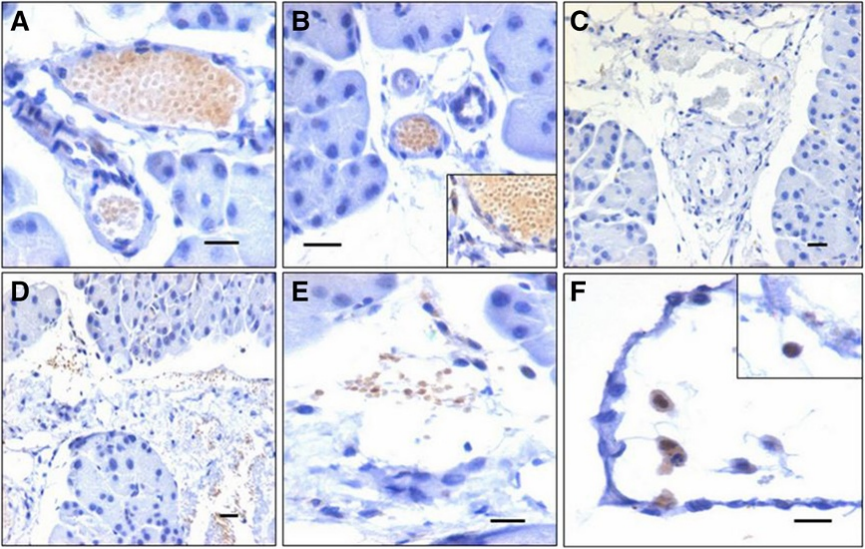
**Immunolocalization of OCT-4 in pancreas on day 5 post pancreatectomy.** The cells in the lumen of blood vessels are positive for OCT-4 **(A)**, **(B)**, a specific marker for pluripotent stem cells. **(C)** Negative control. **(D)** These cells appear to extravasate from the blood vessels. **(E)**, **(F)** OCT-4-positive cells are detected in between newly formed pancreatic lobes as well as below the coelomic epithelium. Few small cells with distinct nuclear OCT-4 are also visualized. Scale bar represents 20 μm.

### Pluripotent VSELs home into mouse pancreas during regeneration

**Figure 4:**
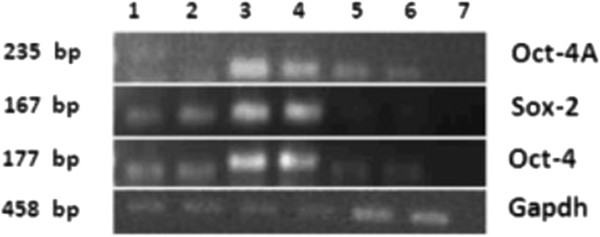
**Reverse transcriptase-polymerase chain reaction results for differential expression of** ***Oct-4*** **and** **Sox-2** **in normal pancreas and after pancreatectomy.** Equal amounts of RNA samples, isolated from normal pancreas and on days 1 and 5 after pancreatectomy, were subjected to reverse transcriptase-polymerase chain reaction (RT-PCR) for pluripotent markers (*Oct-4* and *Sox-2*) and *Oct-4* (inclusive of all transcripts including *Oct-4B*, which is expressed in immediate descendants of very small embryonic-like stem cell ‘progenitors’ when they initiate differentiation) and housekeeping gene transcript glyceraldehyde 3-phosphate dehydrogenase (*Gapdh*). RT-PCR products are electrophoresced for day 1 post pancreatectomy in lanes 1 and 2, for day 5 post pancreatectomy in lanes 3 and 4, and for normal pancreas in lanes 5 and 6; negative control in lane 7. Despite Gapdh being relatively higher in normal pancreas, expression of *Oct-4A, Sox-2* and *Oct-4* is maximal on day 5. Results are in agreement with our histology and immunolocalization data showing migration of stem cells into the pancreas on day 5.

The cells that homed into the pancreas were studied for the expression of OCT-4, a pluripotent stem cell marker that is expressed in the nuclei of VSELs and in the cytoplasm of the progenitors that arise from the VSELs and undergo further differentiation into tissue-committed cell types in a similar manner as we have earlier reported in the ovary and testis [23]. These cells expressed OCT-4 and were present in large numbers in the lumen of blood vessels (Figure 3A,B). These OCT-4-positive stem cells appear to extravasate from the blood vessels/capillaries into the tissue (Figure 3D,E) and expressed either nuclear or cytoplasmic OCT-4. OCT-4-positive cells were also observed just below the coelomic epithelium covering the pancreas surface (Figure 3F). The pluripotent stem cells infiltrating the damaged pancreas post pancreatectomy compared with normal pancreas were studied by reverse transcriptase-polymerase chain reaction analysis for pluripotent transcripts *Oct-4A, Oct-4* and *Sox-2* (Figure 4). The *Oct-4A, Oct-4* and *Sox-2* mRNA transcripts show an increase on day 5 compared with day 1 and normal pancreas.

**Figure 5:**
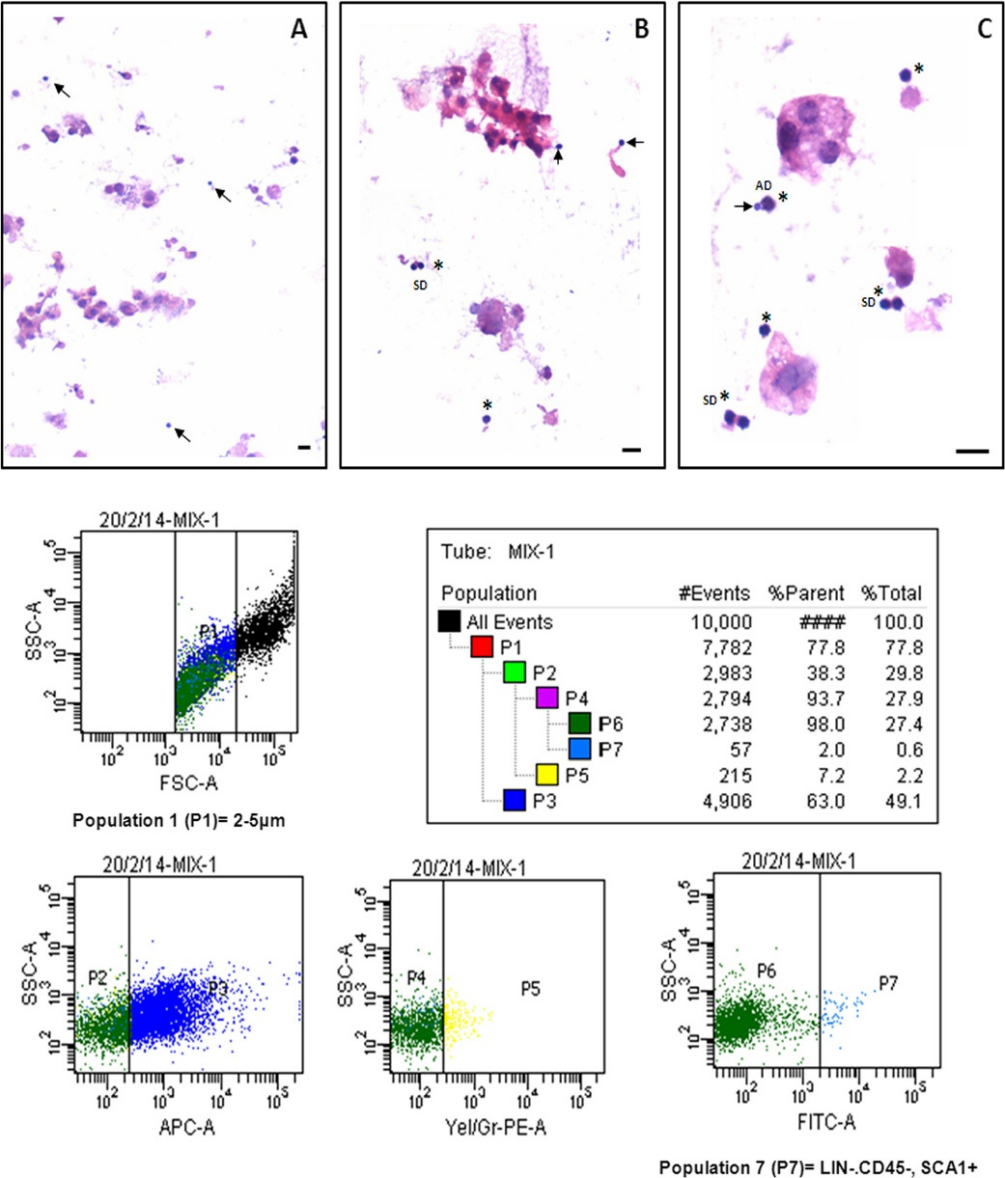
**Very small embryonic-like stem cells in hematoxylin and eosin-stained smears of cell suspension obtained from normal pancreas.** Very small embryonic-like stem cells in hematoxylin and eosin-stained smears of cell suspension obtained from normal pancreas at **(A)** 10×, **(B)** 20× and **(C)** 40× to give an idea of their very small size (arrow), because of which they have eluded researchers to date. Note that these cells have a distinct spherical shape, dark-stained nucleus, high nucleocytoplasmic ratio and minimal cytoplasm. Compared with the stem cells, somatic cells are bigger in size, have abundant cytoplasm and have relatively pale stained nuclei. Two distinct sizes of stem cells can be appreciated at higher magnification **(C)**. The bigger stem cells undergo symmetrical cell division (SD), whereas very rarely we see a very small embryonic-like stem cell (VSEL) giving rise to a bigger stem cell by asymmetric cell division (AD). AD needs to be further characterized. Scale bar represents 20 μm. Flow cytometry analysis shows the presence of 2 to 5 μm VSELs that are LIN^–^/CD45^–^/SCA-1^+^ and comprise 0.6 ± 0.06% of pancreatic cells. APC, allophycocyanin; FITC, fluorescein isothiocyanate; FSC, forward scatter; PE, phycoerythrin; SSC, side scatter.

### Searching for VSELs in normal pancreas

We studied the presence of VSELs in normal mouse pancreas. H & E-stained smears showed the presence of VSELs characterized by their very small size, spherical shape, high nucleocytoplasmic ratio and intensely stained nuclei (Figure 5A,B,C). They could be easily distinguished from the bigger somatic cells with abundant cytoplasm and pale-stained nuclei. The VSELs studied and reported by our group in testis, ovary, cord blood and bone marrow, umbilical cord tissue show similar appearance after H & E staining [22, 23]. The cell suspension obtained after enzymatic digestion was also studied by flow cytometry for the presence of 2 to 5 μm VSELs that are LIN^–^/CD45^–^/SCA-1^+^, as described in the available literature [7]. Briefly cells in the size range of 2-5 µm were gated according to the strategy shown in Figure 5. LIN negative population (P2) was gated from P1 population representing cells in the size range of 2-5 µm. Further CD45 negative cells (P4) were gated from P2. Finally SCA-1 positive cells (P7) were gated from P4. Experiments run in triplicate reveal that 0.6 ± 0.06% of pancreatic cell suspension comprises VSELs (Figure 5).

### Pluripotent VSELs differentiate into PDX-1-positive progenitors that further give rise to both exocrine and endocrine cells

**Figure 6:**
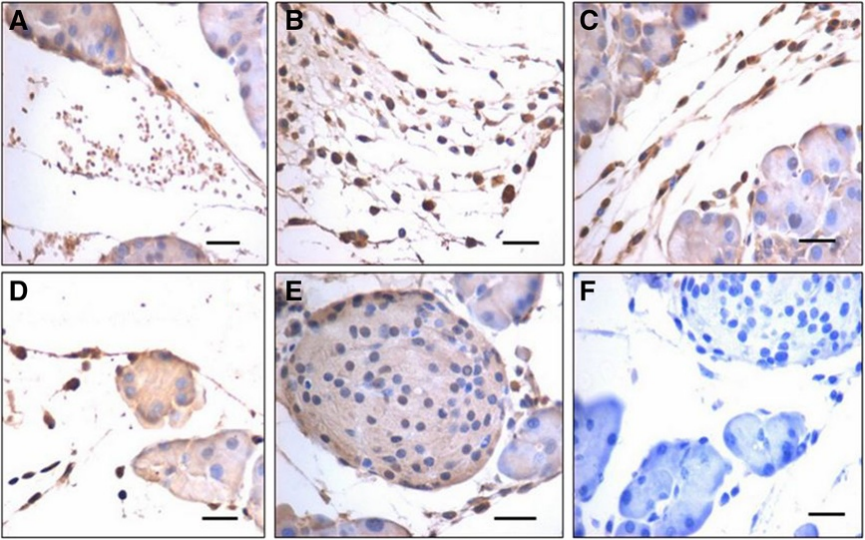
**Immunolocalization of PDX-1 in pancreas sections post pancreatectomy. (A)** Few of the cells that extravasate from the blood vessels express PDX-1. **(B), (C)** These cells appear to migrate in large numbers towards the coelomic epithelium. PDX-1-positive cells are of varying shapes and size including few spherical cells with nuclear PDX-1. Varying size reflects migration of cells and also different stages of differentiation. **(D)** PDX-1-positive progenitor cells are observed along the coelomic epithelium. These cells differentiate into acinar cells along the coelomic epithelium. As these cells differentiate PDX-1, expression shifts from nuclear to cytoplasmic compartment and also staining intensity decreases. This suggests that PDX-1 protein that was initially required as a nuclear transcription factor eventually shifts to the cytoplasm and will eventually get degraded. **(E)** A newly assembled islet also shows faint positive staining for PDX-1. **(F)** Negative control. Scale bar represents 20 μm.

**Figure 7:**
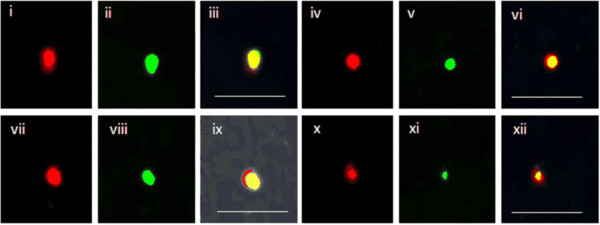
**Colocalization of OCT-4 and PDX-1.** Colocalization of OCT-4 and PDX-1 in four different cells **(i, iv, vii and x)** on smears prepared from pancreatic tissue collected on day 5 post pancreatectomy. Both OCT-4 (red) **(i, iv, vii, x)** and PDX-1 (green) **(ii, v, viii, xi)** colocalize in the nuclei and thus yellow merged images are obtained **(iii, vi, ix, xii)**. It is interesting to note that although PDX-1 is restricted to the nucleus, in places OCT-4 also shows cytoplasmic localization **(vii and ix)**. Results confirm the presence of PDX-1-positive and OCT-4-positive pancreatic progenitors on day 5 after pancreatectomy. PDX-1-positive cells showing the presence of nuclear-to-cytoplasmic OCT-4 shows that they are derived from the nuclear OCT-4-positive very small embryonic-like stem cells. Scale bar represents 20 μm.

PDX-1 is a transcription factor with dual roles including as a differentiation factor and a master switch for expression of genes required for pancreatic development, expressed in both the cellular compartments during embryogenesis, and as a regulator of islet biology in mature islet cells in adults, and its expression is restricted to beta and delta cells [27, 28]. Pancreatectomy results in pancreatic regeneration that recapitulates embryonic development and is associated with PDX-1 expression in BrdU-positive cells [29].Two distinct sizes of cells are observed after extravasation, including very small cells (Figure 6A) with nuclear OCT-4 and relatively bigger cells (Figure 6B,C) that coexpress OCT-4 and PDX-1. These cells appear to migrate towards the coelomic epithelium covering the pancreas. In the vicinity of blood vessels, a fraction of the cells were positive for PDX-1; however, as these cells migrate towards the coelomic epithelium, they changed shape from distinct spherical to fibroblast like and were strongly positive for PDX-1 (Figure 6B,C,D). The acni cells (Figure 6D) and also the cells comprising the newly formed islets (Figure 6E) expressed cytoplasmic PDX-1. PDX-1 and OCT-4 showed colocalization by confocal imaging (Figure 7), thus confirming that OCT-4-expressing stem cells possibly give rise to PDX-1-positive progenitors.

## Discussion

The present study establishes that pancreatic regeneration has a stem cell basis and that neogenesis of islets does occur, contradicting available literature. Xiao and colleagues found no evidence of beta-cell neogenesis in adult pancreas [2]. Unequivocal evidence for the presence of pluripotent VSELs in adult pancreas and their involvement in regeneration post pancreatectomy is provided. VSELs exist in small numbers in normal pancreas (0.6 ± 0.06% of pancreatic cells), but after partial pancreatectomy large numbers are mobilized into the pancreas where they are involved in regeneration. VSELs 2 to 5 μm that are LIN^–^/CD45^–^/SCA-1^+^ and express *Oct-4* and *Sox2* differentiate into PDX-1-positive progenitors that further give rise to both acinar and islet cells.

Increased trafficking of VSELs and mesenchymal stem cells was reported in the peripheral blood of patients with pancreatic cancer compared with healthy controls [13]. Scarlett and colleagues earlier suggested that bone marrow-derived stem cells may be involved during regeneration of mouse pancreas [30]. Two groups have also described earlier the presence of two distinct populations of cells including small and slightly bigger OCT-4-positive cells in human pancreas [31, 32]. Zhao and colleagues detected stem cell markers *Oct-4, Sox 2* and *CD34* in islet-enriched fractions of all 25 adult human pancreas samples, with no significant differences between endocrine and exocrine cell fractions [31]. Immunohistochemical staining for OCT-4, SOX-2, CD133, CD34, CK19, insulin and nestin on human pancreas sections showed that the majority of OCT-4-positive cells were found in the walls of small ducts. Similar localizations were observed for SOX-2-positive cells. The majority of SOX-2-expressing cells were found to coexpress OCT-4, but not *vice versa*. The majority of OCT-4-positive cells had cytosolic staining, while a small number (approximately 1.6%) of cells showed nuclear OCT-4. White and colleagues found nuclear coexpression of pluripotent markers OCT-4/SOX2/NANOG in proliferative islet survivor cells and also in adult human pancreas as well as islet fraction samples [32]. Various techniques such as confocal microscopy, flow cytometry and also western blotting were used to confirm the results. These cells were very small in size (1.5 to 3 μm), resembling VSELs. Expression of OCT-4 in human pancreas-derived primary cell cultures has also been reported [33, 34]. Several groups have thus suggested the presence of OCT-4-positive stem cells in mouse and human pancreas and the results of the present study are in agreement with these earlier reports.

It is rather surprising that despite available reports reviewed above in humans as well as in mice, VSELs have evaded the biologists for so long during so many studies undertaken on pancreatic regeneration after pancreatectomy. Recently, the group of Irvine Weissman, a leading stem cell biologist from Stanford University, created a controversy [35] when they denied the presence of VSELs in mouse bone marrow [36] in agreement with three earlier publications [37–39]. Ratajczak’s group enumerated various factors [12, 40, 41] that could have led to erroneous results by the Stanford group and others. During the present study, we knew what to look for in the pancreatic tissue sections (after pancreatectomy), having worked and published extensively on ovarian, testicular, cord blood and bone marrow VSELs for the last 5 years [22, 23]. Melton’s group used elegant lineage tracing studies to show that adult pancreatic islets are formed by re-duplication rather than stem cell differentiation, and also concluded on the power of the lineage tracing approach over histological snapshots to determine the origin of a cell type [42]. But the histological snapshots in our study have provided novel information on pancreatic regeneration provided one knows what to look for! An alternative explanation to the observed results and conclusions made by Dor and colleagues [42] is possible based on the present study results and VSEL biology. A careful examination of their methods shows that five injections of tamoxifen were given (4 mg, intraperitoneal or subcutaneous twice a week) over a period of more than 15 days that resulted in nuclear translocation of CreER protein, allowing expression of human placental alkaline phosphatase in insulin-expressing cells as well as their progeny. But during this time of more than 2 weeks, VSELs (expected to also harbor the transgene) could also have differentiated into human placental alkaline phosphatase expressing beta cells. Results of present study clearly show that islets are newly formed in the new pancreatic lobes within 5 days (Figure 1J). Thus, rather than re-duplication of pre-existing islets to produce new beta cells (after 15 days of tamoxifen injections) during adult life and after pancreatectomy as concluded by Melton’s group, the new islets could also regenerate from pluripotent VSELs. The present study also highlights the importance of coelomic epithelium during regeneration. The acinar cells appear to differentiate along the coelomic epithelial layer covering the pancreas surface (Figure 1H,I). Li and colleagues reported that differentiation of embryonic pancreas in the presence of mesenchyme, but an absence of coelomic epithelium, led to decreased acinar tissue differentiation [43].

No study is complete in itself and rather provides newer avenues for further research. Studies are underway in our laboratory to understand why VSELs fail to regenerate the islets in a diabetic pancreas and whether these stem cells could be manipulated to cure diabetes. It has been earlier documented that pancreatectomy of STZ-treated pancreas results in regeneration and also restores the euglycemic state in mice [25, 26]. We have also found that STZ treatment does not affect VSELs as suggested by the expression of pluripotent transcripts in the pancreatic tissue (DB, unpublished data, 2014). Huang and colleagues have earlier reported mobilization of VSELs from the bone marrow that home into the STZ-treated mouse pancreas associated with a several-fold increase in pancreas-specific markers (110-fold increase in *Nkx6.1*, eightfold to 10 fold increase in *Pdx-1* and Ptf-1), and also *Sdf-1* (a chemokine involved in mobilization) mRNA was upregulated [44]. Further, transplantation of EGFP^+^ bone marrow cells in STZ mice lowered blood glucose significantly but hyperglycemia reappeared after 84 days. The increased expression of pancreas specific transcripts in bone marrow observed by Huang and colleagues is not surprising, but rather speaks for the potential of VSELs. Being pluripotent by nature, VSELs have the ability to differentiate into any cell type, depending on the body’s requirements. Specific markers are expressed and reported in bone marrow and peripheral blood depending on the type of disease; for example, Nkx-1 and Pdx-1 are upregulated in response to pancreatic damage [44], germ cell-specific markers are expressed when gonadal function is compromised by treatment with busulphan in ovary [45] as well as in testis [46], and neural markers are elevated in the case of stroke [47]. This potential of VSELs to differentiate into specific progenitors depending on their somatic microenvironment and the requirement of the body needs to be appreciated and further explored. Transplantation of beta cells or progenitors obtained after differentiation of ES cells has shown considerable promise [48], but the beneficial effect has remained short lived. Few groups also report an advantage of using mesenchymal cells to improve symptoms [49–51]. All of these varied attempts by the scientific community are not able to outsmart Mother Nature to cure diabetes and we need to pause and deliberate on various available options with an open mind.

Tabar and Studer have recently reviewed the advances and existing shortcomings of translating pluripotent ES and iPS cells to the clinic [52]. They discuss the associated costs, regulatory requirements, scale-up, good manufacturing practice-compliant cultures and related genetic and epigenetic issues. Most importantly, they summarize that the functional status of differentiated cells obtained from pluripotent stem cells resembles their embryonic and fetal counterparts. But the main aim of regenerative medicine is to replace diseased cells in adults with healthy, fully functional cells. The few trials undertaken using human ES cell-derived pancreatic progenitors in mice show only short-term benefit. Against this background, VSELs are autologous, pluripotent stem cells present in adult organs and have no associated ethics, and the present study shows their potential to regenerate adult pancreas. We thus propose that an urgent need exists to develop strategies to manipulate VSELs within the body to our advantage rather than expanding and then differentiating stem cells in a dish. The presence of VSELs in very low numbers should not deter us because they give rise to progenitors that divide rapidly (clonal expansion) in large numbers.

**Figure 8:**
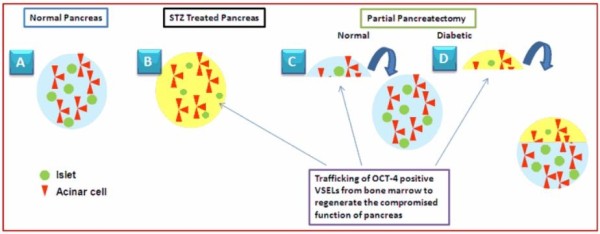
**Schematic representation of stem cell–niche interaction and pancreatic biology. (A)** A healthy niche (blue) in normal mouse pancreas allows the stem cells (very small embryonic-like stem cells (VSELs) and bigger OCT-4 and PDX-1 coexpressing progenitors, please refer to Figures 5C and 7) to function normally and result in normal homeostasis and function. **(B)** Stem cells are unable to regenerate the diabetic streptozotocin (STZ)-treated mouse pancreas because the niche is disrupted (yellow), although they get mobilized into the diseased pancreas in large numbers [44]. **(C)** After partial pancreatectomy, the VSELs quickly regenerate the pancreas and normal function is restored by formation of a healthy niche, acinar and islet cells. **(D)** Mobilized VSELs possibly regenerate the STZ-treated mouse pancreas after partial pancreatectomy including a healthy niche – thus diabetic symptoms are also alleviated, as already reported in the literature [25, 26]. Note normal islets in the regenerating region compared with small sized islets due to STZ treatment. This is a hypothetical model and is open to further discussion and modifications.

We hypothesize that compromised pancreatic function due to loss of beta cells resulting in insulin dependency with age may indeed not be due to lack of stem cells but rather a defect in the microenvironment that prevents the stem cells undergoing normal differentiation into islets and thus presents the symptoms (Figure 8). Indeed, stem cells migrate in large numbers in mouse pancreas after islets are destroyed by STZ treatment [44] but are unable to differentiate and regenerate the islets. As stem cell biologists, it is tempting for us to draw analogies between age-related menopause due to compromised ovarian function and failure of the stem cell niche [53] and diabetes due to compromised pancreatic function with age. Thus it becomes essential to provide a healthy niche along with the stem cells in the diabetic pancreas to reverse the symptoms and to obtain long-term regeneration. The reason for alleviation of diabetic symptoms after partial pancreatectomy of STZ-treated mice can also be explained on the basis of stem cell–niche interaction (Figure 8). Whether a similar mechanism will be functional in humans remains to be seen, but a recent report by Menge and colleagues provides hope since they conclude that partial pancreatectomy results in recovery of glucose control and significant improvement of beta-cell function in humans [54]. Improvement of the pancreatic niche may also be the explanation why transplanting mesenchymal cells is able to improve diabetic symptoms [49–51]. Besides their immunomodulatory ability, mesenchymal cells possibly help restore the microenvironment in the diabetic pancreas and act as a source of growth factors and cytokines that may enable endogenous VSELs to function normally (that is, differentiate into islets), similar to our recent study where transplanting mesenchymal cells in otherwise germ cells depleted testes was able to restore function of VSELs to undergo spermatogenesis [55]. Studies are underway to determine the exact location of VSELs in the pancreas, and what constitutes the niche for pancreatic VSELs needs to be dissected out. The importance of the niche in advancing regenerative medicine was recently highlighted [56, 57].

To conclude, detection of VSELs *in situ* in normal adult pancreas and their involvement during regeneration in the present study offers a practical option for regenerative medicine by manipulating the endogenous (autologous) pluripotent VSELs to treat diabetes in future in addition to other pluripotent stem cells (ES cells or iPS cells) grown in a Petri dish.

## Conclusions

The present study further substantiates the presence of a novel population of VSELs in adult mouse pancreas. Evidence is generated to show that regeneration of pancreas involves stem cells. VSELs regenerate adult mouse pancreas and thus may be an ideal candidate for cell therapy compared with ES/iPS cells, which give rise to the fetal counterparts and thus may be of little value to treat age-related diabetes due to loss of islet function. Further studies are required to understand why VSELs (although present in increased numbers) are unable to regenerate STZ-treated mouse pancreas. The study provides an altogether new perspective to the field of stem cell therapy for diabetes.

## Abbreviations

CD45: marker for hemato-lymphoid cells
ES: embryonic stem
H & E: hematoxylin and eosin
iPS: induced pluripotent stem
LIN: lineage markers for mature hematopoietic cells
OCT-4: marker to define pluripotent state
PDX-1: pancreas duodenum homeobox-1
SCA-1: marker for mouse VSELs and hematopoietic stem cells
SOX-2: marker to define pluripotent state
STZ: streptozotocin
VSEL: very small embryonic-like stem cells.
